# Total hip arthroplasty or hemi-arthroplasty for displaced femoral neck fractures

**DOI:** 10.4103/0019-5413.73653

**Published:** 2011

**Authors:** Mohit Bhandari

**Affiliations:** *Orthopaedic Research Center, Division of Orthopaedic Surgery, Hamilton, Ontario*

## CITATION

Colin Hopley, Dirk Stengel, Axel Ekkernkamp, and Michael Wich. Primary total hip arthroplasty versus hemiarthroplasty for displaced intracapsular hip fractures in older patients: systematic review. BMJ 2010;340:c2332.

## RESEARCH QUESTION

To determine whether total hip arthroplasty (THA) is associated with lower reoperation rates, mortality, general complications, and better function and quality of life than hemi-arthroplasty (HA) for displaced fractures of the femoral neck in patients older than 60 years.

## METHODS

Two independent reviewers conducted a comprehensive search of electronic databases (MEDLINE, EMBASE, the Cochrane register of controlled trials, and publishers’ databases) to identify all available studies. Data were abstracted from all studies that provided sufficient numeric data on at least one of the following end points: reoperation for any cause, dislocation, deep infection, 1 year mortality, general perioperative complications, as well as function and health-related quality of life.

Three independent reviewers assessed the methodologic quality of the studies. Data were pooled using the DerSimonian–Laird random effects model. Funnel plot asymmetry and Egger’s linear regression tests were used to evaluate the presence of publication bias. Differences between results from randomized controlled trials and retrospective cohort studies were identified via stratified analysis, discriminating further between randomized trials with and without proper concealment of treatment allocation. A 2-way sensitivity analysis was used to assess the impact of individual patient and study criteria. Treatment effects between independent subgroups were also compared, providing *P* values and ratios of relative risk (RR) with 95% confidence intervals (CIs).

## FINDINGS

The electronic literature search, and an additional search of the reference lists, uncovered 3843 studies, 15 of which were considered relevant; 4 randomized trials, 3 quasi-randomized trials, and 8 retrospective cohort studies with a total of 1890 patients. When compared with HA, THA was associated with lower risk of reoperation (RR, 0.57; 95% CI: 0.34–0.96). Studies with follow-up intervals greater than 2 years demonstrated larger treatment effects in favor of THA (ratio of RR, 0.44; 95% CI: 0.15–1.26; *P* = 0.13). THA was also beneficial for functional outcomes associated with Harris hip scores (weighted mean difference 5.4; 95% CI: 2.7–8.2). The meta-analysis demonstrated trends toward increased risk of dislocation between treatments (RR, 1.48; 95% CI: 0.89–2.46); however, randomized and quasi-randomized studies with balanced patient baseline profiles and follow-up intervals of 2 or more years trended toward a higher risk of dislocation with THA. General complications were shown to occur somewhat more often in THA (RR, 1.14; 95% CI: 0.87–1.48), whereas pooled RR of infection demonstrated no significant difference (RR, 1.27; 95% CI: 0.64–2.51). Similarly, no significant difference was observed for mortality (RR, 0.92; 95% CI: 0.70–1.21).

## RELEVANCE TO ORTHOPEDIC PRACTICE

When compared with HA, THA appears to reduce the risk of reoperation and improve functional outcomes; however, there is a possible higher risk of dislocation and general complications. The current data do not allow for definitive conclusions about possible treatment effects. A sufficiently powered trial is needed to provide more insightful data on the benefit to risk ratio of THA as compared with HA for displaced femoral neck fractures.

## EXPERT OPINION

The choice of THA in elderly patients with displaced hip fractures is really a tradeoff between improved function and increased risk of hip dislocation. Ultimately, patient care should be individualized and risks and benefits of both procedures presented to patients. Currently, the evidence remains inconclusive as to the definitive benefit of THA in this patient population.

**Evidence summary prepared by:** Sarah Culgin, McMaster University.

